# The Plant Fatty Acyl Reductases

**DOI:** 10.3390/ijms232416156

**Published:** 2022-12-18

**Authors:** Xuanhao Zhang, Yi Liu, Asma Ayaz, Huayan Zhao, Shiyou Lü

**Affiliations:** 1 State Key Laboratory of Biocatalysis and Enzyme Engineering, School of Life Sciences, Hubei University, Wuhan 430062, China; 2 Hubei Hongshan Laboratory, Wuhan 430070, China

**Keywords:** fatty acyl reductases, cuticular wax, taproot wax, suberin, sporopollenin, biosynthesis, regulation

## Abstract

Fatty acyl reductase (FAR) is a crucial enzyme that catalyzes the NADPH-dependent reduction of fatty acyl-CoA or acyl-ACP substrates to primary fatty alcohols, which in turn acts as intermediate metabolites or metabolic end products to participate in the formation of plant extracellular lipid protective barriers (e.g., cuticular wax, sporopollenin, suberin, and taproot wax). FARs are widely present across plant evolution processes and play conserved roles during lipid synthesis. In this review, we provide a comprehensive view of FAR family enzymes, including phylogenetic analysis, conserved structural domains, substrate specificity, subcellular localization, tissue-specific expression patterns, their varied functions in lipid biosynthesis, and the regulation mechanism of FAR activity. Finally, we pose several questions to be addressed, such as the roles of FARs in tryphine, the interactions between transcription factors (TFs) and FARs in various environments, and the identification of post-transcriptional, translational, and post-translational regulators.

## 1. Introduction

Long-chain (LC), or very-long-chain (VLC), primary fatty alcohols are important derivatives of long-chain fatty acids (LCFAs), or very-long-chain fatty acids (VLCFAs). They are primarily involved in the formation of the following four extracellular lipid-phenolic protective layers in the plant kingdom: cuticle coatings in aerial surfaces of land plants, sporopollenin found in the outer walls of pollen spore coatings, suberin which exists in the extracellular walls of various external and internal tissue layers, and suberin-associated waxes in mature taproots [1]. With one exception, primary fatty alcohols are present in the seeds of the jojoba plant (*Simmondsia chinensis*) in the form of wax esters as a lipid energy reserve for postgerminative development [2,3]. In early 1971, Kolattukudy put forward the conjecture that fatty acyl-CoA reductase and aldehyde reductase synergistically catalyze the synthesis of primary fatty alcohols [4]. Up to now, this two-step process via an aldehyde intermediate has not been confirmed in plants. However, it was found that the reduction of fatty acyl-CoAs to primary fatty alcohols can be performed by a single alcohol-forming FAR without releasing the intermediate fatty aldehyde [5,6]. The first *FAR* gene cloned and characterized came from jojoba [6]. Subsequently, related *FAR* genes have been cloned from other plant species, including *Arabidopsis thaliana* [7,8,9,10,11], *Physcomitrella patens* [12], rice (*Oryza sativa*) [13], wheat (*Triticum aestivum*) [14,15,16,17,18], maize (*Zea mays*) [19], *Aegilops tauschii* [20], *Brachypodium distachyon* [21,22], *Brassica napus* [23], and cotton (*Gossypium hirsutum*) [24].

Alcohol-forming FARs in plants can be divided into two categories according to their subcellular localization: microsomal-localized FAR, and plastid-localized FAR, which take acyl-CoA, acyl-ACP, or both [11,25] as substrates. Microsomal-localized FAR is usually in charge of oil production in seeds and the accumulation of wax and suberin, whereas plastid-associated FAR is primarily involved in the biosynthesis of sporopollenin. Each member of the FAR enzyme family is restricted to a unique lipid metabolic pathway due to differences in substrate specificity, tissue-specific expression pattern, and subcellular localization. In addition, lipid metabolism pathways are incredibly complex biological processes in which many enzymes participate, and the regulatory network is even more intricate. Hence, an in-depth exploration of the function and regulation of *FARs* has far-reaching and immense significance to the genetic improvement of crops. Future research should also focus on new biological functions of FAR genes in lipid synthesis and their regulatory molecular mechanisms at different scales, including post-transcriptional, translation, and post-translational levels. Herein, we present a concise review of the latest research into the FAR family of enzymes and further emphasize newly emerging questions that must be addressed to deepen our comprehension of these crucial enzymes.

## 2. Phylogenetic Analysis

**Figure 1:**
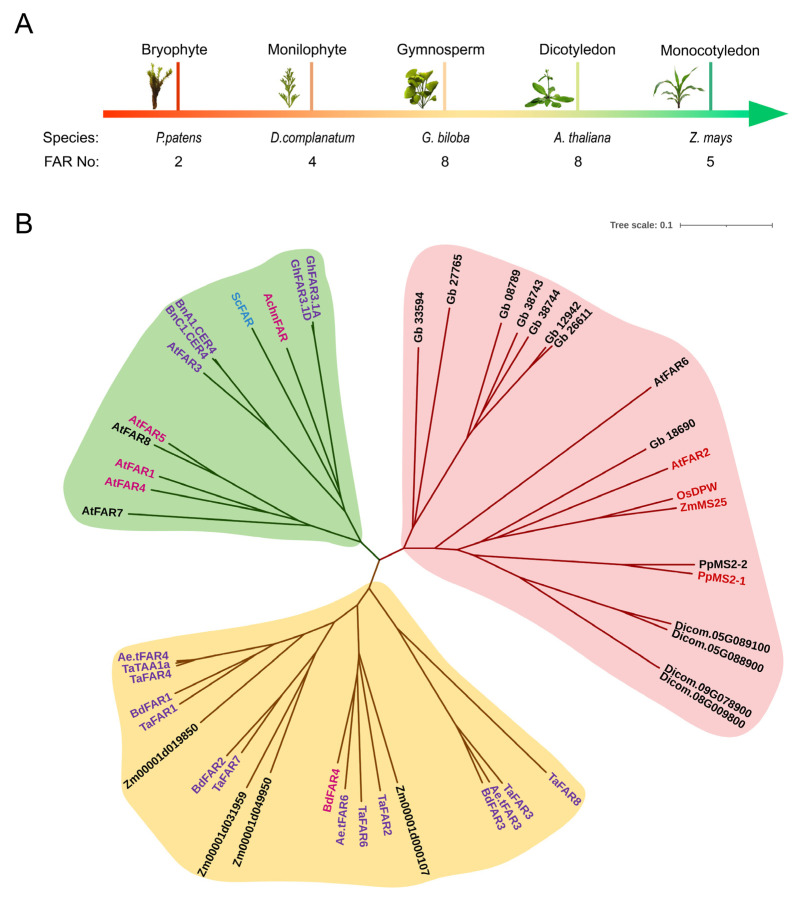
Phylogenetic analysis of plant FARs. (**A**) the number of FAR proteins in the five model species at different stages of terrestrial plant evolution. (**B**) the phylogenetic tree of the FAR members. The analysis involved 49 amino acid sequences from *Arabidopsis thaliana* (At), *Brassica napus* (Bn), *Simmondsia chinensis* (Sc), *Actinidia chinensis Planch* (Achn), *Gossypium hirsutum* (Gh), *Ginkgo biloba* (Gb), *Oryza sativa* (Os), *Zea mays* (Zm), *Physcomitrella patens* (Pp), *Diphasiastrum complanatum* (Dicom), *Triticum aestivum* (Ta), *Aegilops tauschii* (Ae.t), *Brachypodium distachyon* (Bd). FARs with different colors represent distinct functions. Royal purple is associated with cuticular wax, mauve is associated with suberin, red is associated with sporopollenin, blue is associated with storage wax, and black represents an unknown function. The phylogenetic analysis was conducted by MEGA11.0 software using the neighbor-joining method. The tree is drawn proportionally, and the branch length is the same as the evolutionary distance unit used to infer the phylogenetic tree.

*FAR* is reported to be a small plant gene family [1]. To provide some clues about the function of this gene family, five model species with annotated genomes from the evolution of terrestrial plants were selected. These included one bryophyte (*P. patens*), one pteridophyta (*Diphasiastrum complanatum*), one gymnosperm (*Ginkgo biloba*), one dicotyledon (*A. thaliana*), and one monocotyledon (*Z. mays*) (Figure 1A). Then the protein sequences of eight AtFARs were used as templates to perform BLASTPs against all of the genes annotated in the remaining four representative genomes. Phylogenetic analysis using multiple alignments of protein sequences from the five model species of FARs, and the characterized FARs with known functions (Table 1), were inferred using the neighbor-joining method [26]. The consequent neighbor-joining tree showed that all proteins could be clustered into three distinct clades (represented by red, green and yellow, respectively) (Figure 1B). All of the FAR members of the yellow clade originated from monocotyledons, while all of the FAR members of the green clade descended from dicotyledons. Their function is responsible for the biosynthesis of suberin or cuticular wax. The red clade includes the FAR members from the above five model species and rice. Some of the FARs, whose functions have been characterized are required for spore (pollen) outer wall development, including PpMS2-1 from *P. patens*, OsDPW [13] from rice, ZmMs25 [19] from maize, and AtMS2/FAR2 [8] from *Arabidopsis*. These findings suggest that the sporopollenin synthesis-associated FARs existed in both early divergent land plants and the Angiosperms, and the function may be conserved across terrestrial plants. Further analysis with denser sampling and more sophisticated evolution models is helpful to decipher the evolution of FAR.

## 3. Characteristics of Plant FARs

### 3.1. Structural Domains

**Figure 2:**
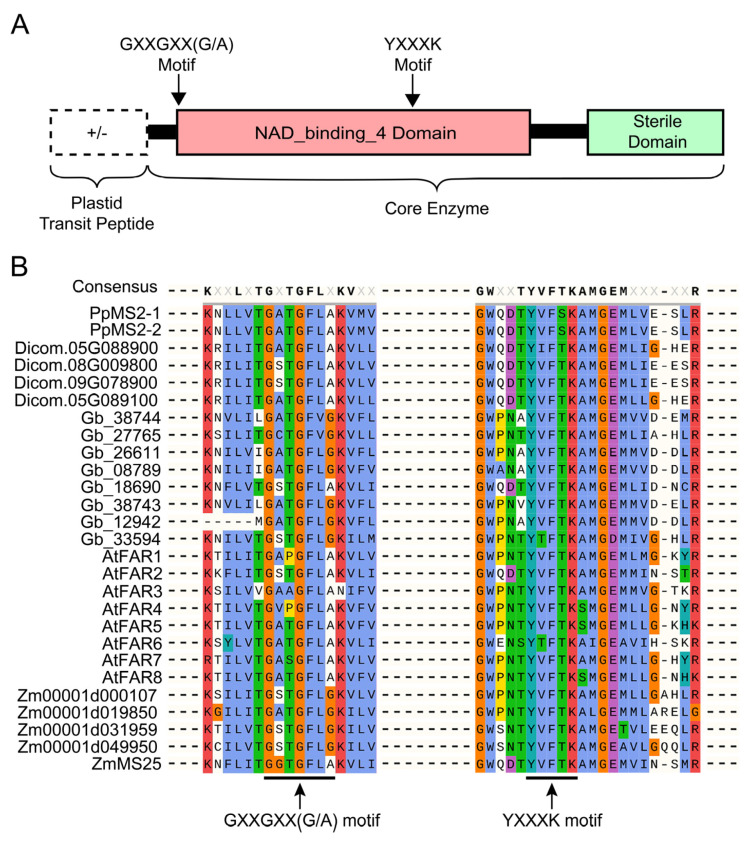
FAR proteins structural domains and protein sequence alignment of FARs from five model species. (**A**) Schematic representation of the structural domains of FAR proteins. NAD_binding_4 domain at the N−terminus is indicated in red, FAR_C domain at the C−terminus is indicated in green. (**B**) Multiple alignments of FAR proteins. The two conserved motifs (GXXGXX(G/A) and YXXXK, X represent any amino acid) within the NAD_binding_4 domains are indicated by black arrows.

Plant FARs are composed of about 500 amino acids in which microsomal-localized FARs contain core enzyme structure composed of NAD_binding_4 domain and sterile domain, whereas plastid-localized FARs contain an N-terminal extension (plastid transit peptide) in addition to core enzyme structure (Figure 2A) [8,11,13,19]. Multiple sequence alignment was carried out for the amino acid sequences of FARs of the five model species mentioned above and the results showed that the NAD_binding_4 domain, of all FARs, contained the NAD(P) H-binding motif (GXXGXX(G/A)) and the active site motif (YXXXK) (Figure 2B), indicating that these two motifs were highly conserved during the evolution of terrestrial plants. A study discovered that constructs containing MS2 fragments with deletion of the NAD_binding_4, or FAR_C domain, or even with deletion of the GXXGXX(G/A) or YXXXK motif, were unable to rescue the phenotype of defective pollen exine in *ms2* mutant [8]. Tyrosine (Y) and lysine (K) residues in the YXXXK active site motif were predicted to play direct roles in the enzyme activity based on kinetic studies with other reductases [31,32]. Site-specific mutations of the two amino acid residues of FAR5 resulted in the inability to produce primary fatty alcohols in yeast [27]. Moreover, subsequent research showed that the mutation of the four amino acid residues (GXXGXX(G/A) and YXXXK, residues underlined) in the above two conserved motifs had a significant impact on the enzymatic activity and substrate selection of ZmMS25 in vitro [19]. 

### 3.2. Substrate Specificity

FARs possess distinct substrate specificities regarding acyl chain saturation and chain length. FAR isoform divergence in substrate specificity is directly connected to their diversity in function and varying subcellular localizations. The physiological properties of the final biosynthetic product are frequently dependent on the substrate specificity of FARs (Table 1). In a fascinating example, the preference of FAR enzymes expressed in pheromone glands for fatty acyl substrates containing cis or trans double bonds leads to reproductive segregation between the two races of European corn borer moth [33].

Despite only 8 FAR members in *Arabidopsis*, AtFARs exhibit varied substrate specificities. AtFAR1, AtFAR4 and AtFAR5 are primarily responsible for the production of C22:0-, C20:0-, and C18:0-OH *in planta*, respectively [10]. Heterologous expression of AtFAR1 in yeast mainly produces C18:0- and C22:0-OH, while expression of AtFAR4 primarily leads to the production of C18:0- and C20:0-OH [10]. When expressed in yeast, AtFAR5 and AtFAR8 produce almost exclusively 18:0- and 16:0-OH, respectively, and amino acids at positions 355 and 377 are essential for dictating 16:0-CoA versus 18:0-CoA chain length specificity. The exchange of amino acids at two particular positions can also convert the substrate specificity of these two proteins [27]. AtMS2/FAR2 is characterized in vitro by the specific use of C16:0-ACP instead of C16: O-CoA to produce C16:0-OH [8], but subsequent studies demonstrated that this enzyme could utilize both C16:0-ACP and C16:0-CoA to generate C16:0-OH [25], which is similar to the substrate specificity of AtFAR6 [11]. Bacteria expressing AtMS2/FAR2 can form C14:0-, C16:0-, and C18:1-OH [9]. AtFAR3/CER4 produces C24:0- and C26:0-OH when expressed in yeast, which is in agrees favorably with the previously established wax profiles of *atcer4* mutants [7,34,35,36]. In addition, AtFAR3 can also take monounsaturated VLCFA-CoAs produced by AtCER17/ADS4 as substrates to synthesize monounsaturated primary alcohols (i.e., C26:1-, C28:1-, and C30:1-OH) in *Arabidopsis* stems [37].

The substrate specificity of the FAR enzymes has also been studied extensively in plant species other than *Arabidopsis*. C16:0- and C18:1-OH are produced when jojoba ScFAR is expressed in *E. coli*, while C22:1-OH is detected when expressed in the seeds of rapeseed (*B. napus*) [6]. Subsequent studies confirmed that jojoba ScFAR had the highest activity toward 18:0-CoA in vitro, followed by 20:1- and 22:1-CoA [28]. TaTAA1a from wheat, an anther-special gene, produces C18:1-, C20:1-, C22:1-, C24:0- and C26:0-OH expressed in mature transgenic tobacco seeds, but it produces C14:0-, C16:0- and C18:1-OH when expressed in *E. coli* [18]. The concern is that the substrate specificity of homologous FAR proteins may also be discrepant concerning the substrate range and preference. OsDPW from rice, an ortholog of AtMS2/FAR2, exhibits more than 270-fold higher specificity for C16:0-ACP than for C16:0-CoA as a substrate [13]. ZmMs25 from maize, also an ortholog of AtMS2/FAR2, catalyzes the reduction of three types of fatty acyl-CoAs (i.e., C12:0-, C16:0- and C18:0-CoA), and has a higher catalytic activity with C12:0-CoA than with C16:0- and C18:0-CoA [19]. Furthermore, four conserved residues (G101, G104, Y327, and K331) of ZmMs25 play an essential role in substrate selection [19]. Additionally, the substrate specificity of homologous FAR proteins may also differ when the substrate has branched chains. BnA1.CER4 and BnC1.CER4 from *B. napus*, the orthologs of AtCER4, appear to prefer branched-chain substrates [23].

### 3.3. Subcellular Localization and Expression Pattern

In the plant kingdom, FAR proteins are confined to only two subcellular compartments (i.e., plastid and ER) (Table 1). Several pollen development-associated FARs are known to localize to the plastid envelope, including AtMS2/FAR2 [8], OsDPW [13], and ZmMs25 [19], whereas those wax and suberin-associated FAR enzymes are reported to localize in the ER where wax and suberin biosynthesis occurs. 

The expression pattern of a gene is closely related to its function (Table 1). *AtFAR1*, *AtFAR4*, and *AtFAR5* are mainly expressed in tissues where the suberin deposits [10]. *AtFAR3*/*CER4* is highly expressed in aerial organs of the plant, which is consistent with its roles in wax biosynthesis [7], in addition, the *FARs* from other plants also display similar expression patterns, such as *Ae.tFAR3*, *Ae.tFAR4,* and *Ae.tFAR6* from *Ae. tauschii* [20], *GhFAR3.1A* and *GhFAR3.1D* from cotton [24], and *TaFARs* from *Triticum aestivum* [14,15,16,17]. *AtMS2*/*FAR2* expression is restricted to flowers, which is consistent with its roles in pollen exine development [8]. In addition to *Arabidopsis*, *PpMS2-1* from *P. patens* exhibits a sporophyte-specific expression pattern [12]. *ZmMs25* is expressed specifically in anther, which is in agreement with its roles in anther and pollen development in maize [19]. Rice *OsDPW* is mainly expressed in the tapetum and microspores [13]. Further study of the expression pattern of *FAR* in diverse plant species is required to clarify the function of *FAR* during lipid metabolism comprehensively.

## 4. The Function of FAR in Extracellular Lipid Synthesis

### 4.1. Cuticular Wax Synthesis-Associated FARs

**Figure 3:**
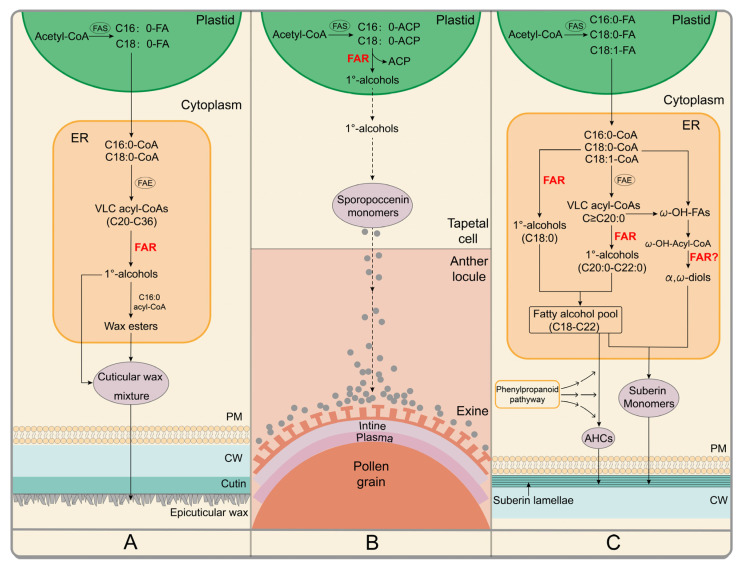
Pathways of FARs involved in lipid metabolism in the plant kingdom. (**A**) ER-Localized FARs are involved in the accumulation of cuticular waxes. The biosynthesis of waxes begins with C16 or C18 de novo fatty acid synthesis in the plastid. Then, utilizing C16 and C18 acyl-CoAs as substrates, the fatty acid elongase (FAE) complex performs a reiterative cycle to synthesize saturated VLCFAs. These VLCFAs are further modified into primary alcohols and wax esters. (**B**) Plasmid-Localized FARs are involved in sporopollenin biosynthesis. Acyl-ACPs synthesized de novo in the plastid are reduced by FAR to produce fatty alcohols. This product could then be exported to the anther locule by an unknown mechanism where it polymerizes at the surface of the microspore. (**C**) ER-Localized FARs are involved in the suberin and suberin-associated wax production. De novo fatty acid synthesis occurs in the plastid. Fatty acyl elongation occurs via the FAE complex producing VLCFAs. FARs catalyze acyl reduction to produce suberin monomer primary alcohols and α, ω-diols. Coumaric, caffeic, and ferulic acids produced by the phenylpropanoid pathway are linked to fatty alcohols by BAHD-type acyltransferases to produce alkyl hydroxycinnamates (AHCs). Abbreviations: PM, plasma membrane; CW, cell wall.

Cuticular wax is a complex mixture of VCLFAs and their derivatives ranging from C20 to C60 synthesized in the ER (Figure 3A), They include primary alcohols, fatty aldehydes, alkanes, and esters, and may also contain cyclic compounds, such as terpenoids and sterols [38,39] on the aerial surface of all terrestrial plants which plays a vital role in protecting them from the attack of diverse biotic and abiotic stress factors, such as drought, UV-B radiation, mechanical damage, and even bacterial and fungal pathogens [38,40,41,42]. Changes in the cuticular wax primary alcohol composition significantly impact the crystal structure and hydrophobic properties of the epidermis [43,44]. Cuticular wax primary alcohols can also act as signal molecules and play an important role in pathogen and host recognition [45]. In addition, triacontanol (C30-OH) acts as a growth regulator, enhancing plant photosynthesis and increasing dry matter accumulation [46].

*AtFAR3*/*CER4* plays a dominant role in the accumulation of cuticular wax-associated primary alcohols of *Arabidopsis* [7]. Intuitively, the *atcer4* mutant shows a stem “glossy” phenotype, suggesting that the absence of primary alcohols has a significant impact on the assembly and arrangement of epidermal wax crystals [7,34]. Interestingly, the mutation of *AtFAR3/CER4* results in the almost complete deletion of VLC monounsaturated primary alcohols in the stems in comparison to the wild type, and co-expressing AtFAR3/CER4 with AtCER17/ADS4 in yeast produced VLC monounsaturated (n-6) primary alcohols, indicating VLC monounsaturated acyl-CoAs are also the substrates of AtFAR3/CER4 [37].

Wax-associated FAR enzymes have been extensively studied in plant species other than *Arabidopsis*. These FAR enzymes include eight TaFARs (TaFAR1-TaFAR8) from wheat [14,15,16,17], three Ae.tFARs (Ae.tFAR3, Ae.tFAR4, and Ae.tFAR6) from *Ae. Tauschii* [20], three BdFARs (BdFAR1, BdFAR2, and BdFAR3) from *B. distachyon* [21], one CsCER4 from cucumber (*Cucumis sativus*) [47], and two BnFARs (BnA1.CER4 and BnC1.CER4) from *B. napus* [23]. Most are involved in the biosynthesis of straight-chain primary alcohols, while both BnA1.CER4 and BnC1.CER4 are involved in the biosynthesis of *iso*-branched primary alcohols in cuticular waxes.

The primary alcohols and esters generated by the alcohol-forming pathway only account for 15–25% of the total wax in *Arabidopsis* inflorescence stems and rosette leaves. In contrast, the alcohols take a predominant role in leaf epidermal wax in some important crops, such as in corn and barley where primary alcohols account for about 70–80% of the wax components [48,49,50]. Therefore, an accurate interpretation of each FAR’s function in synthesizing cuticular wax primary alcohols among different crop species is crucial for reconstructing plant cuticular wax layers in some important crops.

### 4.2. Sporopollenin Synthesis-Associated FARs

Sporopollenin, a complex polymer consisting of polyhydroxylated aliphatic compounds and phenolics, has extreme stability and recalcitrance, thus ensuring the integrity of the pollen when it is subjected to various external physical and chemical pressures such as hydrostatic, chemical reagents, and non-oxidative chemical and biological degradation [51,52,53]. De novo synthesis of fatty acids occurs in tapetal plastids, where they are reduced to LC primary alcohols by FAR proteins (Figure 3B).

To date, sporopollenin synthesis-associated *FAR* genes were studied in several plant species such as *Arabidopsis*, rice, and maize [8,13,19]. *AtMS2/FAR2* from *Arabidopsis* is first identified as essential for sporopollenin synthesis [8,54]. *OsDPW* from rice [13] and *ZmMs25* from maize [19] are also required for sporopollenin biosynthesis, suggesting that the metabolic pathway of sporopollenin is conserved among angiosperms. Interestingly, unlike the *Arabidopsis atms2* mutant, the anther cuticle of the rice *dpw* mutant is also defective, which indicates that the functions of related genes and/or enzymes have diversified during evolution [13]. In addition to angiosperms, sporopollenin is also widely found in *Chlorophyta*, *Bryophyta*, *Pteridophyta*, *Marchantia polymorpha,* and even fungi [55]. Moreover, *PpMS2-1*, a putative moss homolog of *AtMS2/FAR2*, participates in the development of the outer wall of the spore since its mutant phenotype is remarkably similar to that of defective microspore exine in *Arabidopsis* [12]. These findings indicate that the underlying mechanism of sporopollenin biosynthesis is highly conserved during the land plant evolutionary process. Moreover, during the process of evolution from lower plants to higher plants, the composition of the spore outer wall (pollen outer wall) becomes more complex [12].

### 4.3. FARs Involved in Suberin and Suberin-Associated Waxes Biosynthesis

Suberin is a hydrophobic heteropolymer composed of phenolics, glycerol, and various fatty acid derivatives that mainly act as a protective barrier for controlling the flow of water, solutes, and gases, protecting plants from various abiotic stresses and pathogenic infections [56,57,58,59,60]. Its aliphatic portion is a polyester composed mainly of ω-Hydroxyl fatty acids, α, ω- dicarboxylic acid with chain lengths ranging from C16 to C28, FAs, and primary fatty alcohols [61] (Figure 3C).

In *Arabidopsis*, *AtFAR1*, *AtFAR4,* and *AtFAR5* are reported to be involved in the accumulation of suberin-associated primary alcohols, and the total fatty alcohol load in suberin is reduced by 70–80% in *atfar1 atfar4 atfar5* triple mutant lines [10,60]. In *B. distachyon*, the mutation of *BdFAR4* leads to a significant reduction in the content of C20:0- and C22:0-OH compared with the wild type [22].

In the periderm of underground storage organs, suberin is found in association with waxes. These suberin-associated waxes are composed of linear aliphatic with shorter chain lengths than cuticular wax and have been found in diverse plant species such as potato (*Solanum tuberosum*) [62], Camelina (*Camelina sativa*) [63] and *Arabidopsis* [64,65]. Alkyl hydroxycinnamates (AHCs), which are formed by esterification of C18:0 to C22:0 primary fatty alcohol with coumaric acid, caffeic acid, or ferulic acid, are the main component of suberin-associated waxes [65] (Figure 3C). The biosynthesis of AHCs of suberin-associated root waxes includes the following steps: the biosynthesis of hydroxycinnamate, the reduction of fatty acyl-chains, and the transfer of CoA-activated hydroxycinnamate derivatives onto hydroxylated aliphatic [66]. In *Arabidopsis*, three *FARs* (*AtFAR1*, *AtFAR4,* and *AtFAR5*) required for primary alcohol synthesis in suberin are also involved in the production of fatty alcohols in suberin-associated taproot waxes [60,65,67]. The contents of soluble fatty alcohols and AHCs in root waxes of *atfar1 atfar4 atfar5* triple mutant lines are reduced by more than 80% [60,67]. Apart from *Arabidopsis*, AHC synthesis-associated *FAR* is rarely reported in plant species. However, AHCs are widely present in angiosperms [62,68], gymnosperms [69,70], and possibly even in *P. patens* [71], suggesting that some enzymes might play similar roles as AtFARs in catalyzing the production of AHCs.

## 5. Regulation of *FAR* Genes

Extracellular lipid protective barriers are crucial in the tolerance to various environmental stresses. Many of the *FAR* genes involved in extracellular lipid metabolism are induced by various abiotic or biotic stresses including drought, salt, cold, wounding, and the infection of fungi. For example, the transcriptional levels of three suberin-associated genes, *AtFAR1*, *AtFAR4,* and *AtFAR5*, are gradually up-regulated after wounding and salt treatment [10]. The transcripts of several wax-associated genes including *TaFARs* (i.e., *TaFAR1*-*TaFAR8*) and *BdFARs* (*BdFAR1*-*BdFAR4*) are also induced by abiotic stress treatment such as cold, drought, and/or high salt [14,15,16,17,21,22]. The transcripts of *TaFAR6*, *TaFAR7,* and *TaFAR8* are also induced by powdery mildew (*Blumeria graminis*) infection [17]. Thus far, some *MYB* transcription factors have been identified to regulate the expression levels of wax-associated *FARs* under abiotic stresses (Table 2). For example, *AtMYB94* was dramatically induced by salt stress and drought stress, and it can activate the expression of *AtFAR3*/*CER4* through direct promoter binding [72]. *PtoMYB142* from *Populus tomentosa* contributes to drought tolerance by directly binding to the promoter of the wax biosynthesis gene *PtoCER4* and regulating its expression [73]. 

In addition, some TFs were identified to play positive roles in regulating the expression levels of suberin-associated genes (Table 2). In the seed coat, *AtMYB107* interacts strongly with the *AtFAR1* promoter, and its mutation significantly reduces the expression of *AtFAR1*, *AtFAR4*, and *AtFAR5* [74]. In the cell wall of *Arabidopsis* leaf epidermal cells, *AtMYB41* overexpression increases the abundance of *AtFAR1*, *AtFAR4,* and *AtFAR5* transcripts and leads to the ectopic deposition of suberin monomer C18-C22 primary alcohols [75]. *BdMYB41* from *B. detachyon*, which is closely related to *AtMYB41*, directly interacts with the promoter region of *BdFAR4* [22]. During wound suberization, *AchnMYB41*, *AchnMYB107*, and *AchnMYC2* from kiwifruit activate *AchnFAR* to enhance primary fatty alcohol accumulation [30]. Some highly conserved MYBs are found to regulate the sporopollenin synthesis-associated *FAR* genes (Table 2). *AtMYB103* (also called *MYB80* and *MS188*) and its direct upstream regulator *AtAMS* are identified to be essential for the expression of *AtMS2/FAR2* in pollen walls [76,77]. TaTDRL and TaMYB103 are homologs of AtAMS and AtMYB103, respectively. Both can directly bind to the promoter to synergistically activate the expression of TaTAA1a [78]. OsMYB80 and ZmMYB84, as homologs of AtMYB103, directly activate the expression of OsDPW and ZmMs25, respectively [19,79]. Thus far, most studies have focused on the roles of *MYBs*, whereas only one study showed that *Arabidopsis SQUAMOSA PROMOTER BINDING PROTEIN-LIKE 9* (*SPL9*) indirectly regulates *AtCER4* expression by affecting other unknown TFs [80].

**Table 2:** Transcription factors associated with *FARs* regulation.

Transcription factors	Species	Regulatory target	Associated metabolic pathway *in planta*	Reference
*AtMYB94*	*Arabidopsis thaliana*	*AtFAR3/CER4 * ^1^	Cuticular wax biosynthesis	[72]
*AtSPL9*	*Arabidopsis thaliana*	*AtFAR3/CER4 * ^2^	Cuticular wax biosynthesis	[80]
*AtMYB39*	*Arabidopsis thaliana*	*AtFAR1*^1^, *AtFAR4*^1^ and *AtFAR5* ^1^	Suberin biosynthesis	[81,82]
*AtMYB107*	*Arabidopsis thaliana*	*AtFAR1*^1^, *AtFAR4*^3^ and *AtFAR5* ^3^	Suberin biosynthesis	[74]
*AtMYB41*	*Arabidopsis thaliana*	*AtFAR1*^3^, *AtFAR4*^3^ and *AtFAR5* ^3^	Suberin biosynthesis	[75]
*AtMYB80/MYB103/MS188*	*Arabidopsis thaliana*	*AtMS2/FAR2 * ^1^	Sporopollenin biosynthesis	[76,77]
*PtoMYB142*	Populus tomentosa	*PtoCER4 * ^1^	Cuticular wax biosynthesis	[73]
*AchnMYB41*, *AchnMYB107,* and *AchnMYC2*	*Actinidia chinensis Planch*	*AchnFAR * ^1^	Suberin biosynthesis	[30]
*BdMYB41*	*Brachypodium distachyon*	*BdFAR4 * ^1^	Suberin biosynthesis	[22]
*TaTDRL* and *TaMYB103*	*Triticum aestivum*	*TaTAA1a * ^1^	Pollen exine development	[78]
*OsMYB80*	*Oryza sativa*	*OsDPW * ^1^	Sporopollenin biosynthesis	[79]
*ZmMYB84*	*Zea mays*	*ZmMs25 * ^1^	Sporopollenin biosynthesis	[19]

^1^ Direct regulation through promoter binding; ^2^ Indirect regulation through other transcription factors; ^3^ No experimental data exists to confirm whether it is direct regulation or indirect regulation.

## 6. Conclusions and Perspectives

Fatty acyl reductases target acyl-CoAs or acyl-ACPs to provide fatty alcohol substrates for lipid synthesis processes which is vital for the normal growth and development of plants. Herein, a brief cluster analysis was first conducted on the related FAR proteins and their conserved structural domains, tissue-specific expression patterns, subcellular localization, and unique roles in different lipid metabolic pathways. These were then summarized in this review (Figure 3). Lastly, this review also described the mechanisms by which FAR is regulated. Although the progress made in recent decades has significantly advanced our understanding of the *FAR* gene family, particularly the conservation of function and regulation, several questions remain unanswered.

1. The pollen wall is a complex multi-layer structure wrapped on the outer surface of pollen (Figure 3B). Aliphatic alcohols not only exist in the exine in the form of sporopollenin but also in the cavities of the pollen exine in the form of tryphine [83]. Tryphine is composed of complex lipids, wax esters, flavonoids, hydroxycinnamoyl spermidine metabolites, and proteins [84,85]. Little is known about the formation of tryphine. Therefore, it is of great interest to investigate whether any specific alcohol-forming *FARs* are involved in tryphine production.
2. In *Arabidopsis*, AtFAR1, AtFAR4, and AtFAR5 display different specificity towards substrates with different chain lengths, which are mainly responsible for the synthesis of C22:0-OH, C20:0-OH, and C18:0-OH, respectively. Interestingly, recent studies showed that the levels of LC suberin monomers including C18:0-OH positively correlate with environmental factors such as precipitation, evapotranspiration, temperature, and UV index, whereas those of VLC suberin monomers, including C20:0-OH and C22:0-OH, display the opposite trend [86]. This indicated that *AtFAR1*, *AtFAR4,* and *AtFAR5* are differentially regulated by various environmental cues. Understanding the regulatory mechanism of *AtFAR1*, *AtFAR4,* and *AtFAR5* in response to different environmental conditions will provide new insights into plants’ abilities to adapt to different environmental factors.
3. Thus far, regulatory mechanisms of *FARs* have been comprehensively studied at the transcriptional level, but little is known about how *FARs* are regulated at the post-transcriptional level, the translational level, and the post-translational level.

## Figures and Tables

**Table 1:** Substrate specificity, subcellular localization, expression pattern and function of FARs.

Species	Protein	Accession No.	Substrate specificity *in planta*	Subcellular localization	Expression pattern	Functional association *in planta*	Reference
*Arabidopsis thaliana*	AtFAR1	NP_197642	22:0-CoA	Unidentified	Expressed in various organs, highly expressed in young roots, rosette leaves, and flowers	Suberin and taproot waxes	[10]
	AtMS2/FAR2	NP_187805	16:0-CoA, 16:0-ACP ^1^	Plastid	Flower-specific expression	Sporopollenin	[8,25]
	AtFAR3/CER4	NP_567936	24:0-, 26:0-, 28:0-, 30:0-CoA	ER ^5^	Expressed in various organs, highly expressed in aerial organs	Cuticular waxes	[7]
	AtFAR4	NP_190040	20:0-CoA	Unidentified	Mainly expressed in young and mature roots	Suberin and taproot waxes	[10]
	AtFAR5	NP_190041	18:0-CoA	Unidentified	Mainly expressed in young and mature roots	Suberin and suberin-associated waxes	[10,27]
	AtFAR6	NP_191229	16:0-CoA, 16:0-ACP ^1^	Plastid	Mainly expressed in stems epidermis	Might provide functional redundancy to AtFAR2	[11]
	AtFAR7	NP_197634	Unidentified	Unidentified	Stigmas-specific expression	Likely a pseudogene	[9]
	AtFAR8	NP_190042	16:0-CoA ^2^	Unidentified	Stigmas-specific expression	Unidentified	[27]
*Simmondsia chinensis*	ScFAR	AAD38039	18:0-, 20:1-, 22:1-CoA ^1^	Unidentified	Unidentified	Seed storage energy	[6,28]
*Physcomitrella patens*	PpMS2-1	NC_037259	Unidentified	Unidentified	Highly expressed in the sporophyte	spore wall	[12]
*Oryza sativa*	OsDPW	ABF94174	16:0-ACP	Plastid	Mainly Expressed in the Tapetum and Microspores	Sporopollenin	[13]
*Zea mays*	ZmMs25/MS6021	NC_050104	12:0-, 16:0-, 18:0-CoA ^1^	Plastid	Specifically expressed in anthers from stages 8b-9 to 9-10, with the peak at stage 9-10	Sporopollenin	[19,29]
*Triticum aestivum*	TaFAR1	KF926683	22:0-CoA ^2^	ER	Highly expressed in seedling leaf blades and anthers	Cuticular waxes	[14]
	TaFAR2	KJ675403	18:0-CoA ^2^	ER	Low-level expression in aerial organs	Cuticular waxes	[16]
	TaFAR3	KT963076	28:0-CoA ^2^	ER	Widely expressed in aerial organs, highly expressed in seedling leaves	Cuticular waxes	
	TaFAR4	KT963077	24:0-CoA ^2^	ER	Widely expressed in aerial organs, highly expressed in seedling and flag leaves	Cuticular waxes	
	TaFAR5	KJ725345	22:0-CoA ^2^	ER	Highly expressed in leaf blades, anthers, pistils, and seeds	Cuticular waxes	[15]
	TaFAR6	MF804951	24:0-, 26:0-CoA ^2^	ER	Highly expressed in the seedling leaf blades	Cuticular waxes	[17]
	TaFAR7	MF817443	24:0-, 26:0-CoA ^2^	ER	Highly expressed in the seedling leaf blades	Cuticular waxes	
	TaFAR8	MF817444	24:0-CoA ^2^	ER	Highly expressed in the seedling leaf blades	Cuticular waxes	
	TaTAA1a	CAD30692	18:1-, 20:1-, 22:1-, 24:0-, 26:0- CoA ^4^	Unidentified	Specifically expressed in the sporophytic tapetum cells	Pollen wall	[18]
*Brachypodium distachyon*	BdFAR1	ASK86469	22:0-CoA ^2^	ER	Highly expressed in early developing leaves, leaf sheaths, nodes, and internodes	Cuticular waxes	[21]
	BdFAR2	ASK86470	26:0-CoA ^2^	ER	Mainly expressed in leaf sheaths, nodes, internodes, and early-developing leaves	Cuticular waxes	
	BdFAR3	ASK86471	26:0-CoA ^2^	ER	Highly expressed in leaves at 40 d, leaf sheaths, and internodes	Cuticular waxes	
	BdFAR4	QTK16914	20:0-, 22:0-CoA ^2^	ER	Root-specific expression	Suberin	[22]
*Gossypium hirsutum*	GhFAR3.1A	XP_016744016	Unidentified	Unidentified	Highly expressed in leaves and rapidly elongating fibers	Cuticular waxes	[24]
	GhFAR3.1D	XP_016753267	Unidentified	Unidentified	Highly expressed in leaves and rapidly elongating fibers	Cuticular waxes	
*Actinidia chinensis Planch*	AchnFAR	PSS03141	18:0-, 20:0-, 22:0-, 24:0-CoA ^3^	Unidentified	Highly expressed in fruits	Suberin	[30]
*Brassica napus*	BnA1.CER4	AID60102	26:0-CoA ^2^, precursors with branched chains	ER	Highly expressed in leaves	Cuticular waxes	[23]
	BnC1.CER4	AOS88709	26:0-CoA ^2^, precursors with branched chains	ER	Highly expressed in leaves	Cuticular waxes	
*Aegilops tauschii*	Ae.tFAR1	AMH86041	16:0-CoA ^2^	Unidentified	Low-level expression in various organs	Maybe suberin or sporopollenin	[20]
	Ae.tFAR2	M8B4B3	18:0-CoA ^2^	Unidentified	Low-level expression in various organs	Maybe suberin or sporopollenin	
	Ae.tFAR3	M8BJ01	26:0-CoA ^2^	Unidentified	Highly expressed in seedling leaves and flag leaves	Cuticular waxes	
	Ae.tFAR4	M8CRK2	24:0-CoA ^2^	Unidentified	Widely expressed in aerial organs	Cuticular waxes	
	Ae.tFAR6	M8C929	28:0-CoA ^2^	Unidentified	Low-level expression in various organs	Cuticular waxes	

^1^ Catalytic activity in vitro; ^2^ Catalytic activity in yeast; ^3^ Catalytic activity in tobacco leaves; ^4^ Catalytic activity in tobacco seeds; ^5^ Subcellular localization performed in yeast, needs *in planta* verification.

## Data Availability

Data are available from the authors on request.
